# Nearly free silanols drive the interaction of crystalline silica polymorphs with membranes: Implications for mineral toxicity

**DOI:** 10.3389/fchem.2022.1092221

**Published:** 2023-01-16

**Authors:** Cristina Pavan, Guillermo Escolano-Casado, Chiara Bellomo, Stefania Cananà, Maura Tomatis, Riccardo Leinardi, Lorenzo Mino, Francesco Turci

**Affiliations:** ^1^ Department of Chemistry, University of Turin, Turin, Italy; ^2^ “G. Scansetti” Interdepartmental Centre for Studies on Asbestos and Other Toxic Particulates, University of Turin, Turin, Italy; ^3^ Louvain Centre for Toxicology and Applied Pharmacology, Institute of Experimental and Clinical Research, Université catholique de Louvain, Brussels, Belgium; ^4^ Nanostructured Interfaces and Surfaces Interdepartmental Centre, University of Turin, Turin, Italy

**Keywords:** quartz (SiO_2_), cristobalite, stishovite, silanol (SiOH), surface hydroxyl, membrane, crystalline silica, silica polymorphs

## Abstract

Crystalline silica (CS) is a well-known hazardous material that causes severe diseases including silicosis, lung cancer, and autoimmune diseases. However, the hazard associated to crystalline silica is extremely variable and depends on some specific characteristics, including crystal structure and surface chemistry. The crystalline silica polymorphs share the SiO_2_ stoichiometry and differentiate for crystal structure. The different crystal lattices in turn expose differently ordered hydroxyl groups at the crystal surface, i.e., the silanols. The nearly free silanols (NFS), a specific population of weakly interacting silanols, have been recently advanced as the key surface feature that governs recognition mechanisms between quartz and cell membrane, initiating toxicity. We showed here that the nearly free silanols occur on the other crystalline silica polymorphs and take part in the molecular interactions with biomembranes. A set of crystalline silica polymorphs, including quartz, cristobalite, tridymite, coesite, and stishovite, was physico-chemically characterized and the membranolytic activity was assessed using red blood cells as model membranes. Infrared spectroscopy in highly controlled conditions was used to profile the surface silanol topochemistry and the occurrence of surface nearly free silanols on crystalline silica polymorphs. All crystalline silica polymorphs, but stishovite were membranolytic. Notably, pristine stishovite did not exhibited surface nearly free silanols. The topochemistry of surface silanols was modulated by thermal treatments, and we showed that the occurrence of nearly free silanols paralleled the membranolytic activity for the crystalline silica polymorphs. These results provide a comprehensive understanding of the structure-activity relationship between nearly free silanols and membranolytic activity of crystalline silica polymorphs, offering a possible clue for interpreting the molecular mechanisms associated with silica hazard and bio-minero-chemical interfacial phenomena, including prebiotic chemistry.

## 1 Introduction

Crystalline silica (CS) polymorphs are a group of minerals that share the SiO_2_ stoichiometry and differentiate for crystallographic structure and spatial arrangement of the silica structural units. CS, mainly in the form of α-quartz, is ubiquitous on the Earth’s crust in rocks, sand, biogenic deposits, fly, and volcanic ashes. Quartz is the most stable crystalline form of silica at ambient conditions. Besides quartz, the other polymorphs that are thermodynamically stable in nature as chemically pure CS are cristobalite, tridymite, coesite, and stishovite. Other forms, such as keatite and moganite, exist as mineralogical rarities (Guthrie and Heaney, 1995). After quartz, cristobalite and tridymite are the most abundant forms of CS and are mainly found in silicic volcanic rocks and volcanic ashes (Damby et al., 2016). Cristobalite is also formed during the high-temperature calcination of diatomaceous Earth and used in many industrial processes for the production of abrasives, pigments, metal casting, and chemicals (Nattrass et al., 2015). More recently, cristobalite was reported to be used for the fabrication of artificial stone composite materials (Di Benedetto et al., 2021). Tridymite, which is formed at low-pressure and high-temperature (>870°C), has also been found in high-temperature meteoritic impact settings and extraterrestrial rocks, as in the Gale crater on Mars (Morris et al., 2016). Coesite and stishovite are dense forms of CS and are found in nature when quartz rocks undergo high-temperature and pressure processes, e.g., rocks impacted by meteorites (Guthrie and Heaney, 1995).

Because of its peculiar geophysical and physico-chemical properties, silica has attracted much attention in a variety of scientific disciplines (Rimola et al., 2013; Croissant et al., 2020; He et al., 2021; Ma et al., 2022). However, inhalation of respirable crystalline silica (RCS), i.e., silica particles with an aerodynamic diameter lower than 4 μm (Brown et al., 2013), is associated with severe lung diseases, which include silicosis, lung cancer, and autoimmune diseases (Iarc, 2012; Leung et al., 2012; Cullinan et al., 2017; Hoy and Chambers, 2020). Workplace exposure to RCS particles may occur in many anthropogenic activities, encompassing mining, excavation, digging, drilling, and sanding. Due to its large industrial usage, quartz pathogenic effects have been largely investigated and an extensive scientific literature is available on the toxicological mechanisms of quartz (Mossman and Churg, 1998; Kawasaki, 2015; Borm et al., 2018; Pavan et al., 2020). In parallel, the pathogenic effects of the other silica polymorphs were investigated, and cristobalite and tridymite were also showed to induce fibrosis in the lungs of rats (King et al., 1953; Wiessner et al., 1988). Following those pioneer studies, the International Agency for Research on Cancer (IARC) classified quartz and cristobalite as human lung carcinogens (Iarc, 2012). Cristobalite and quartz particles evidenced similar mechanisms of toxicity in experimental models of lung cells (Mossman and Glenn, 2013). Both polymorphs activated the inflammasome, induced the release of the pro-inflammatory cytokine IL-1β and of the basic fibroblast growth factor (bFGF) from macrophages (Peeters et al., 2014), and triggered similar profiles of chemokines and cytokines secretion in bronchial epithelial cells (Perkins et al., 2012). *In vivo* experiments demonstrated similarities in intensities and mode of action (MoA) of these two polymorphs. Inhalation studies on rats evidenced similar patterns of pulmonary histopathology and immunological responses (Mossman and Glenn, 2013). Instillation studies showed similar increase of lung permeability, epithelial cell injury, and thoracic lymph node enlargement (Housley et al., 2002). So far, the other polymorphs have been little investigated in toxicological studies. Some pioneer studies indicated that coesite and stishovite were less biologically active than quartz (Brieger and Gross, 1967; Wiessner et al., 1988; Driscoll, 1995), but no indications of the MoA or the molecular determinants that induce toxicity were provided. Since the silica polymorphs only differ for their structural arrangement of silica units, the understanding of the causes of their different toxicological outcomes may shade light on the structure-activity relationship (SAR) that drives silica biological interaction, with consequences for the MoA and biocompatibility profiles of silica, and for metal oxides in general.

Each polymorph is unique in its spacing, lattice structure, and angular relationship of the atoms. The crystallographic structure of quartz is a spiral network of [SiO_4_]^4-^ tetrahedra about the *Z*-axis, in which the tetrahedra are linked to form a hexagonal structure. In cristobalite and tridymite, [SiO_4_]^4-^ are packed in a two and three-layer structure, respectively (Guthrie and Heaney, 1995). Cristobalite and tridymite exhibit a more open structure with respect to quartz and may incorporate larger amounts of impurities, for instance Al^3+^ or Fe^3+^ substituting for Si^4+^, and these substitutions may reduce cristobalite toxicity (Nattrass et al., 2017). Coesite consists in [SiO_4_]^4-^ units that form four-membered rings which are joined in a chain structure. Stishovite is the only silica polymorph in which silicon is octahedrally coordinated by six oxygen atoms. Different crystal structure and crystallographic plane result in different density and distribution of surface silica hydroxyl groups, i.e., silanols (≡Si‒OH), as well as different intersilanol hydrogen (H)-bonding possibilities (Murashov et al., 2006; Musso et al., 2009). As the surface of a xenobiotic represents the dialoging chemical entity which interacts with the surrounding biological molecules, different arrangements and properties of surface moieties may diversify biological responses. Using molecular modelling, Murashov and co-workers (Murashov et al., 2006) predicted the membranolytic activity of several CS polymorphs by computing the densities of surface geminal silanol groups (>Si−(OH)_2_). However, to the best of our knowledge, there are no experimental studies investigating possible SAR between surface topochemistry and the toxicity mechanism of CS polymorphs.

We recently showed that the interaction of quartz particles with biomembranes and the initiation of the toxicity mechanism is regulated by the amount of a specific population of surface silanols, i.e., the nearly free silanols (NFS) (Pavan et al., 2020). This silanol family exhibits a peculiar intersilanol geometry (4–6 Å apart distance) that maximizes the interaction energy with zwitterionic phospholipid head groups and determines membrane damage (Pavan et al., 2022). It has been shown that, when silica is phagocytized by alveolar macrophages and confined into phagolysosomes, damage to the phagolysosome membrane leads to the activation of the NACHT, LRR, and PYD domains-containing protein 3 (NALP3) inflammasome (Dostert et al., 2008; Hornung et al., 2008). Thus, the interaction of quartz particles with membranes has been suggested as the molecular initiating event that triggers the maturation and release of pro-inflammatory and pro-fibrotic mediators contributing to the inflammatory response, which is the common feature underlying silica-associated diseases (Pavan and Fubini, 2017).

This work questions the hypothesis that NFS might occur, in addition to quartz, also on the other silica polymorphs and take part in the molecular interactions that are established between the CS surfaces and cellular membranes. This will prove the existence of a common SAR between the silica polymorph structures and their membranolytic activity. Cristobalite, tridymite, coesite, and stishovite powders were fully characterized for physico-chemical properties of interest, including crystallographic structure, elemental composition, morphology, specific surface area, particle size, and surface charge. The capacity of the CS polymorphs to cause cell membrane damage was assessed using red blood cells (RBCs) as model membranes. RBCs, which are non-phagocytic cells, are a useful proxy to probe the membranolytic activity of mineral particles (Pavan et al., 2019). Surface specific analyses in highly controlled conditions were carried out by infrared (IR) spectroscopy to define the silanol families exposed at CS surface, including the NFS. Thermal treatments were used to tailor the quantity of NFS on cristobalite and stishovite surfaces and the membranolytic activity of these NFS-modified polymorphs was assessed. A commercial quartz that showed toxicity effects (IARC, 2012) and NFS-related membranolytic and inflammatory activity (Pavan et al., 2020) was used as positive reference to comparatively discuss the results.

## 2 Materials and methods

### 2.1 Crystalline silica polymorphs

A commercial sample of finely powdered cristobalite obtained from quartz heated at high temperature (>1,500°C) was kindly supplied by an industrial producer. The raw material was sieved through a 30 µm mesh sieve on a vibrating apparatus before use. Tridymite was kindly provided by Prof. U. Saffiotti (Laboratory of Experimental Pathology, National Cancer Institute, National Institutes of Health, Bethesda, MD; United States). Coesite and stishovite were kindly provided by Prof. W. Stöber (Chemical Industry Institute of Toxicology Research Triangle Park, NC, United States) and obtained by chemical extraction from ores of the Meteor Crater (AZ, United States). These samples have been used in previous studies (Cerrato et al., 1995; Fenoglio et al., 2000). The commercial quartz Min-U-Sil 5 (US Silica Company, Berkely Springs, WV, United States) was used as positive reference particle because of its well-documented membranolytic (Pavan et al., 2020) and toxicity effects (IARC, 2012).

### 2.2 Chemical reagents

When not otherwise specified, all reagents were purchased from Merck (Sigma-Aldrich, Germany). The water used was ultrapure Milli-Q water (Merck-Millipore, Burlington, MA, United States). Red blood cells (RBCs) were obtained from sheep blood in Alsever’s solution (Oxoid, United Kingdom).

### 2.3 Crystallographic structure

Crystallinity was assessed by X-Ray Powder Diffraction (XRPD) in the Bragg-Brentano configuration with a Miniflex 600 (Rigaku, Japan). Spectra were collected in the 2θ range (from 10° to 90°), with a step width of .01°, 1°/min of speed, and Cu Kα radiation at 40 kV and 15 mA. The diffractograms obtained were analyzed with the Rigaku PDXL v.2.8 software.

### 2.4 Particle morphology and elemental composition

The morphology of dry particles was assessed by Field Emission Scanning Electron Microscopy (FE-SEM) with a TESCAN S9000G microscope (Czech Republic) equipped with a Schottky FEG source. Images were taken at various magnifications and accelerating voltages, commonly 10 or 5 KV and 100 pA. Dry particles were deposited on conductive stubs covered with carbon tape and, when required, coated with chrome (5 nm) to prevent the electron beam from charging the sample. Elemental analysis was carried out by Energy Dispersive X-ray Spectroscopy (EDX) on the same microscope using the Oxford Aztec Ultim Max detector (Oxford Instruments, United Kingdom). Spectra were collected on at least twelve region (ca. 650 µm^2^) per sample, at 2–10 k magnification, 10 or 20 KV accelerating voltage, and processed using Aztec suite (v. 4.2, Oxford Instruments, United Kingdom).

### 2.5 Specific surface area (SSA)

The SSA of the particles was evaluated by the Brunauer, Emmett, and Teller (BET) method, using an ASAP 2020 porosimeter (Micromeritics, Norcross, United States). Samples were degassed at 150°C for 2 h before the analysis. Depending on the expected SSA, Kr (SSA <5 m^2^/g) or N_2_ (SSA >5 m^2^/g) adsorption at −196°C was measured. BET surface area was then calculated over the range P/P_0_ = .05–.25 (12 points).

### 2.6 Particle size and surface charge

Particles were dispersed in .01 M PBS (1 mg/mL, pH 7.4) and sonicated for 2 min on ice with an ultrasonic probe (horn, 3 mm; frequency, 20 kHz; maximum power output, 25 W; amplitude, 120 μm; Sonopuls HD 3,100, Bandelin, Germany). The size distribution of micrometric particles was measured by Flow Particle Image Analysis using a FPIA-3000S (detection range .8–160 μm; Malvern Instruments, United Kingdom). Particle dispersions were injected (ca. 5 mL) into the measurement cell, then stirred at 360 rpm to avoid particle sedimentation. Particle images were captured using stroboscopic illumination and a charge-coupled device camera. Data were processed by the Sysmex FPIA software (version 00–13). The hydrodynamic diameter of fine particles (<1 μm) was assessed on diluted dispersions (.1 mg/mL, in order to have an attenuator ∼8) by Dynamic Light Scattering (DLS) using a Zetasizer Nano ZS (detection range 10–1,000 nm; Malvern Instruments, United Kingdom). Data were obtained by three independent measures, three replicates for each measurement. Particle surface charge (ζ potential) was measured by Electrophoretic Light Scattering (ELS) with the Zetasizer Nano ZS.

### 2.7 Free radical release

Generation of hydroxyl radicals (^•^OH) was evaluated in a cell free test by Electron Paramagnetic Resonance (EPR) spectroscopy coupled with the spin trapping technique, according to well-established protocols developed for silica (Fubini et al., 2004). Samples (5%) were suspended in .01 M PBS containing 25 mM 5,5-Dimethyl-1-Pyrroline-N-Oxide (DMPO; Cayman Chemical, United States) as spin trapping agent. Generation of the [DMPO-OH]^•^ adduct was evaluated after 15 min of incubation (Zhang et al., 2012). EPR spectra were recorded on a Miniscope MS 100 (Magnettech, Berlin, Germany) spectrometer. The instrument settings were: microwave power 10 mW; modulation 1,000 mG; scan range 120 G; center of field 3,330 G. Blanks were performed in the same condition in the absence of sample. TiO_2_ nanopowder (P25, Degussa, Essen, Germany, primary particle size 20 nm, BET surface area 50 m^2^/g) was used as positive control. TiO_2_ suspension (120 μg/mL) was irradiated for 30 min with UV light (365 nm, irradiation intensity 1 mW/cm^2^) by a 100 W UV lamp (FV-97600-15 Cole-Parmer, Paris, France), as previously described (Kose et al., 2021).

### 2.8 Surface silanol distribution

To determine the surface silanol distribution of the particles, measurements were carried by Diffuse Reflectance Infrared Spectroscopy (DRIFT) following a protocol previously described (Pavan et al., 2020). Briefly, a Spectra-Tech diffuse reflectance unit, equipped with an environmental chamber allowing the connection to a conventional vacuum line (residual pressure, ≤1 × 10^−4^ mbar), was used to carry out *in situ* all desorption/adsorption experiments. The samples were analyzed in powder form, with ∼50 mg of silica sample. The spectra were collected with a Bruker Vector22 FTIR spectrometer (Globar source, MCT detector; resolution, 4 cm^−1^) averaging 128 scans for spectrum to obtain a good signal-to-noise ratio. The silica samples underwent an H/D isotopic exchange by adsorption/desorption of D_2_O vapors from a pure solution (Sigma-Aldrich; 99.90% D) in order to convert surface silanols (≡SiOH) in the ≡SiOD form. Spectra were converted in Kubelka-Munk units and normalized for the SSA.

### 2.9 Hemolytic activity

RBCs were purified from sheep blood in Alsever’s solution by centrifugation at 3,000 × g for 2 min (Rotina 380R; Hettich, MA) and washing three times with .9% NaCl. RBCs were suspended in .01 M PBS at the final concentration of 5% by volume. Sheep RBCs were used because they showed a sensitivity to silica very similar to that of human RBCs (Arienzo and Bresciano, 1969). Particles were dispersed in .01 M PBS and sonicated 2 min on ice (horn, 3 mm; frequency, 20 kHz; maximum power output, 25 W; amplitude, 120 μm; Sonopuls HD 3,100, Bandelin, Germany), just before testing. Serial dilutions of the starting particle dispersions were performed according to the final surface area doses used for experiments. Dispersions were distributed in quadruplicate in a transparent 96-well plate (150 µL/well), and the RBC suspension was then added (75 mL/well). Negative and positive controls consisted of .01 M PBS and .1% Triton-X 100 in PBS, respectively. The plate was incubated on a plate shaker at 37°C for 30 min, and then centrifuged at 216 × g for 5 min. Supernatants were transferred to a new plate (75 mL/well), and the absorbance of the hemoglobin released was determined at 540 nm on a UV/vis spectrophotometer (Ensight, Perkin-Elmer, Waltham, MA) using the software Kaleido 2.0 (Perkin-Elmer).

### 2.10 Heat treatments of the particles

To obtain heated particles, 500 mg of the pristine CS polymorphs were heated in a muffle furnace for 2 h at the indicated temperatures (ramp up: 10°C/min) and allowed to cool at room temperature (r.t.) in the same furnace. Samples were stored in glass vials in a glovebox (MBraun LABstar glove box supplied with pure 5.5 grade argon, O_2_ < 1 ppm, H_2_O < 1 ppm) before testing hemolysis and surface silanols. The temperatures and modus operandi were selected based on previous studies on cristobalite (Fubini et al., 1999) and stishovite (Cerrato et al., 1995).

### 2.11 Statistics

Statistical parameters, including the number of independent experiments and statistical significance, are reported in the figures and figure legends. Unless otherwise stated, data are presented as mean ± standard error of the mean (SEM).

## 3 Results and discussion

### 3.1 Physico-chemical characteristics of the crystalline silica polymorphs

**FIGURE 1:**
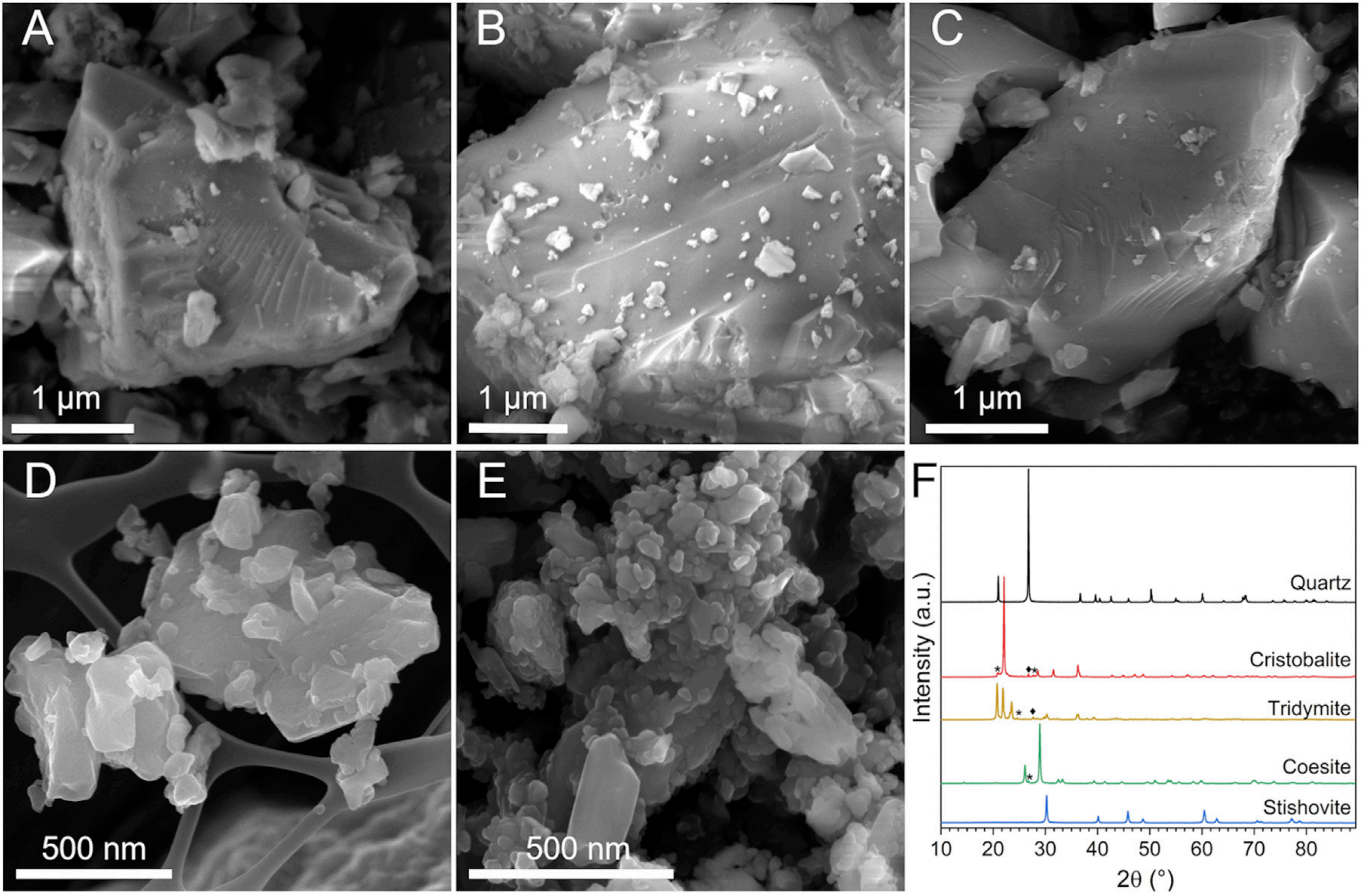
Morphology and crystallinity of the CS polymorphs. **(A–E)** Micrographs obtained by FESEM analysis: **(A)** quartz, **(B)** cristobalite, **(C)** tridymite, **(D)** coesite (background: grid of the stub), and **(E)** stishovite particles. **(F)** XRPD patterns of the CS polymorphs: ♦quartz and *other contaminants.

Quartz, cristobalite, and tridymite exhibited a similar morphology (Figure 1, Supplementary Figure S1), characterized by particles with irregular shapes and acute edges. Sample morphology is typical of CS dust obtained by mechanical fracturing (Turci et al., 2016). Conchoidal fractures, i.e., the curved fractures crossing a series of crystal planes (Pavan and Fubini, 2017), were observed on quartz surfaces (Figure 1A) and on cristobalite (Figure 1B) and tridymite (Figure 1C), less frequently. At the nano scale, conchoidal fractures does not follow specific lattice planes or preferential crystallographic orientation. Instead, a quasi-amorphous layer, the so-called Beilby layer, is formed and is invoked to describe the loss of long-range atomic order at the crystal interface (Turci et al., 2016). These three dusts showed primary particles of a few microns, and a fraction of sub-micrometric particles adhering on the surface of the largest ones.

Coesite (Figure 1D, Supplementary Figure S1D) and stishovite (Figure 1E, Supplementary Figures S1E–F) showed particles with a smoother and roundish surface, a peculiar morphology compared to the CS polymorphs above mentioned. Primary particles were in general smaller than quartz, with an average size of ca. 50–80 nm or larger agglomerates/aggregates of ca. 500 nm, especially stishovite which exhibited a large fraction of ultrafine particles in the nanometric range (Supplementary Figure S1F). The analysis of the specific surface area (SSA) by BET method pointed out the lower SSA of quartz, cristobalite, and tridymite (i.e., SSA ranging from 2 to 5 m^2^/g) with respect to coesite and stishovite (18 and 20 m^2^/g, Table 1). The higher SSA of coesite and stishovite is possibly due to their lower particle size, as porosity is usually negligible for crystalline silica.

**TABLE 1:** Physico-chemical characteristics of the crystalline silica polymorphs.

Sample	SSA (m^2^/g)	Sample composition^c^ (oxide wt% ± SD)	Particle size (µm) ± SD in medium (.01 M PBS)				ς-Potential^f^ (mV, pH 7.4)	^•^OH radical generation^g^
			CE average diam.^d^	D90^d^	D50^d^	Z-average (PdI)^e^		
Quartz	5.0^a^	SiO_2_: 98.8 ± 0.7	1.9 ± 0.9	3.1 ± 0.2	1.6 ± 0.1	—	−27 ± 2.0	absent
		Al_2_O_3_: 1.2 ± 0.7						
Cristobalite	2.3^a^	SiO_2_: 99.9 ± 0.2	1.4 ± 1.3	2.0 ± 0.1	1.1 ± 0.1	—	−26 ± 1.4	absent
		Al_2_O_3_: .1 ± 0.2						
Tridymite	5.2^b, h^	SiO_2_: 100 ± 0.2	1.3 ± 0.6	1.8 ± .03	1.1 ± .02	—	−24 ± 1.1	absent
Coesite	18^b^	SiO_2_: 100 ± 0.3	—	—	—	.70 ± .12 (.23)	−22 ± 1.4	absent
Stishovite	20^b^	SiO_2_: 93.4 ± .7^i^	3.0 ± 2.6	6.5 ± 0.2	2.0 ± 0.1	.66 ± .22 (.59)	−19 ± 1.2	absent
		Al_2_O_3_: 4.1 ± 0.6						
		TiO_2_: 2.5 ± 0.6						

^a, b^ Evaluated by BET method, Kr^a^ or N_2_^b^ adsorption.

^c^Evaluated by SEM-EDS analysis.

^d^Assessed by Flow Particle Image Analysis (FPIA), CE diameter, the diameter of the circle having the same projected area as the particle; D50 and D90, the values at which the cumulative frequency reaches 50 or 90%.

^e^Measured by Dynamic Light Scattering (DLS), the polydispersity index (PdI) is reported in brackets.

^f^Measured by Electrophoretic Light Scattering (ELS) in .01M PBS (pH 7.4).

^g^Measured by Electron Paramagnetic Resonance (EPR) coupled with spin trapping probe (i.e., DMPO).

^h^From (Daniel et al., 1993).

^i^Chlorine contamination due to the mineral extraction procedure (Fahey, 1964; Bohn and Stöber, 1966) was detected (1.41 ± 2.29 wt. %).

Crystallinity and sample purity were verified by XRPD (Figure 1F). Diffractograms of the five mineral samples evidenced both well-crystallized minerals and some traces of quartz as accessory phase in the cristobalite and tridymite samples. The elemental composition analysis was carried out *via* EDX spectroscopy and is reported in Table 1; Supplementary Figure S2. All samples but stishovite were very pure and revealed a SiO_2_ content higher than ca. 99 wt%. Some traces of Al were found in the commercial quartz, as also previously reported (Ghiazza et al., 2013). Stishovite showed the occurrence of some Al_2_O_3_ (ca. 4 wt%) and traces of TiO_2_ and Cl. This latter impurity is presumably due to the chemical extraction of stishovite from the raw material as concentrated HCl was used (Fahey, 1964; Bohn and Stöber, 1966).

The hydrodynamic diameter of the polymorph particles was assessed by flow particle image analysis (FPIA) and dynamic light scattering (DLS), depending on the investigated size range (Table 1, Supplementary Figure S3). Measurements were carried out in phosphate buffer solution (.01 M PBS, pH 7.4), the same milieu used for membranolysis assessment. Quartz, cristobalite, and tridymite exhibited an average circle equivalent (CE) diameter of ca. 1.5 µm, and 90% of particles with average diameter <3.2 µm, which indicates that most of the particles are in the respirable size range (i.e., <4 µm). Cristobalite and tridymite showed 90% of particles with average diameter below 2.0 and 1.8 µm, respectively, which was slightly lower than what observed for quartz particles (3 µm). The standard deviation (SD) associated with the average diameter of cristobalite was higher than that of quartz and tridymite, indicating a slightly higher heterogeneity in size. Coesite and stishovite, assessed by DLS, showed an average hydrodynamic diameter in the submicrometric range, ca. .6–.7 µm. While for coesite particles the quality of the DLS measurements signaled quite homogeneous particle dispersion (polydispersity index—PdI, of ca. 2.3) (Supplementary Figure S3E), stishovite demonstrated a high PdI (>3) which would indicate the occurrence of large particle agglomerates. This was confirmed by FPIA analysis, which revealed the presence of a fraction of micrometric aggregates/agglomerates with an average CE diameter of ca. 3 µm. Images captured by FPIA clearly showed the occurrence of aggregates/agglomerates of submicrometric primary particles for stishovite (Supplementary Figure S4).

The assessment of the ζ potential, measured by electrophoretic light scattering (ELS), showed that all CS polymorphs were negatively charged in PBS at the physiological pH (7.4). Stishovite particles showed ζ potential values slightly lower than the other particles. Negative surface charge on silica at pH 7.4 is mainly due to deprotonation of silanol groups which yield negatively charged silanolates (Si‒O^−^). The surface heterogeneity derived from the structural characteristics of each polymorph may be responsible of the observed slight variations in ζ potential. The different silanol groups—isolated, NFS, geminal, vicinal, H-bonded—which populate the silica surface are all acidic but characterized by different acidity constants (Musso et al., 2011; Sulpizi et al., 2012). Thus, the discrepancy between the net negative surface charge of quartz and stishovite here observed suggests that on quartz some silanol groups are characterized by a higher acidity with respect to the groups present on stishovite.

Moreover, the CS polymorphs were evaluated for their capacity to generate hydroxyl radicals. Reactive oxygen species (ROS), and in particular ^•^OH radicals, may have important implications in the mechanism of silica toxicity, contributing to the cell oxidative stress (Fubini and Hubbard, 2003; Zhang et al., 2012). Assessment of ^•^OH radicals was performed in the same experimental conditions of the membranolysis test and showed that all the CS polymorphs examined aren’t able to generate ^•^OH radicals in buffered suspension (Table 1, Supplementary Figure S5). Therefore, a possible contribution of ^•^OH radicals to the membranolytic activity of the CS samples here investigated should be ruled out, as also shown in previous studies on silica membranolysis (Vallyathan, 1994; Clouter et al., 2001; Pavan et al., 2013).

### 3.2 Membranolytic activity and surface silanol distribution of the crystalline silica polymorphs

**FIGURE 2:**
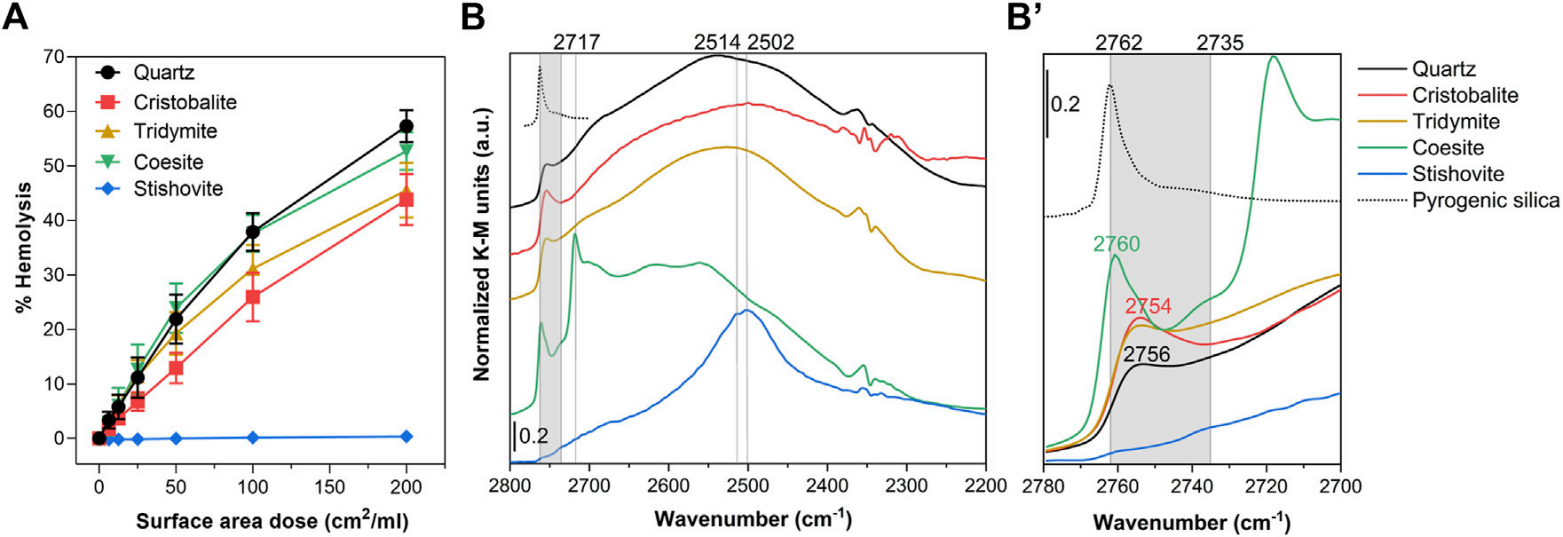
Nearly free silanols (NFS) occur on some crystalline silica polymorphs and are related to their membranolytic activity. **(A)** Membranolytic activity (percent hemolysis) of CS polymorph particles. Increasing surface area doses (0, 6.25, 12.5, 25, 50, 100, and 200 cm^2^/mL) of quartz (positive reference particle), cristobalite, tridymite, coesite, and stishovite were incubated for 30 min with purified sheep RBCs. Values reflect the fraction of the total hemoglobin content released and are reported as mean ± SEM of three independent experiments. **(B)** Surface silanol distribution measured by DRIFT spectroscopy after H/D exchange in the νOD spectral region. **(B’)** Enlarged section of the spectra showing the νOD spectral region assigned to NFS (gray highlight, 2,760 to 2,735 cm^−1^) and the isolated silanol peak of a pyrogenic amorphous silica (Rimola et al., 2018) reported as reference (dotted spectrum, peak at 2,762 cm^−1^).

The membranolytic activity of the panel of CS polymorphs was assessed with RBCs (Figure 2A). Cristobalite, tridymite, and coesite showed a strong dose-dependent hemolytic activity, almost attaining that of quartz used as positive reference particle (IARC, 2012; Pavan et al., 2020), which indicates a strong perturbative activity towards the RBC membrane. Conversely, stishovite wasn’t hemolytic, even at the highest dose. Results are consistent with previous experiments (Stalder and Stöber, 1965; Wiessner et al., 1988), albeit higher doses of material were used in those studies. RBCs play no part in the pathogenesis of silicosis or cancer. However, several studies reported a correlation between the hemolytic activity of several quartz specimens and their lung inflammatory response, supporting the evidence that the lung inflammogenic potential of quartz is driven by its crystal surface properties (Clouter et al., 2001; Duffin et al., 2001; Pavan et al., 2014). The paramount role of the particle surface topochemistry, i.e., the chemistry of the surface reactions that depends on the spatial arrangement and orientation of surface moieties, has become more clear in these last years to explain the inflammatory activity of different quartz sources (Pavan et al., 2020) and of amorphous silica obtained from various synthetic routes (Croissant et al., 2020; Pavan et al., 2020). We recently reported that the membranolytic and inflammatory activity of quartz is determined by NFS, which occurs in variable amount depending on the silica source (Pavan et al., 2020).

To inspect the surface silanol profile of the CS polymorphs and possible occurrence of NFS, we used IR spectroscopy in Diffuse Reflectance (DRIFT) mode after hydrogen-deuterium (H/D) exchange (Figure 2B, Supplementary Figure S6). The sample powder was outgassed for 60 min to remove the multilayer of physisorbed water from its surface. Subsequently, the CS polymorphs were exposed to several adsorption/desorption cycles of D_2_O vapors until reaching spectral invariance, thus, promoting the isotopic exchange and converting the SiOH species in SiOD (Supplementary Figure S6). The isotopic exchange methodology permits to isolate the signals due to the surface species from those of the bulk, as previously reported (Pavan et al., 2020) (Figure 2B, Supplementary Figure S6). The νOD (i.e., O‒D stretching vibration) profile of quartz, cristobalite and tridymite showed a broad band centered at ca. 2,720–2,250 cm^−1^ assigned to silanols mutually engaged in strong H-bonds (Figure 2B). At higher wavenumber the three polymorphs showed a peak in the region of those silanols that experience weak mutual interactions. Slightly different relative intensities and variation in the position of the peak were observed for the three polymorphs (Figure 2B’). These peaks all fall in the region which has been assigned to NFS (ca. from 2,735 cm^−1^ to 2,760 cm^−1^) (Pavan et al., 2020). Conversely, fully isolated, non-interacting silanols (>6 Å apart) generate a different νOD signal which is evidenced by the sharp peak centered at 2,762 cm^−1^ (Figure 2B’) (Rimola et al., 2018). Coesite showed a narrow component centered at 2,760 cm^−1^, which indicates that some NFS also occur on this sample. Compared to the other polymorphs, coesite showed also some sharp and intense bands at lower νOD, in the region assigned to H-bonded silanols.

Stishovite exhibited a strong complex band, which is the superimposition of two components, one at 2,514 and the other at 2,502 cm^−1^, positioned in the region assigned to silanols mutually engaged in strong H-bonds. NFS or isolated silanol groups were non-detected on this sample. A similar hydroxyl pattern for stishovite was formerly reported by a study from our laboratory (Cerrato et al., 1995), albeit the adopted methodology did not allow to discriminate between inner and surface hydroxyl species. This atypical silanol pattern may be assigned to the octahedral coordination of Si atoms (similar to the rutile structure), which differ from the regular tetrahedral coordination of all the other CS polymorphs. This structure results in a surface characterized by densely packed OH that mutually interact *via* strong H-bonds. The peculiar silanol configuration of stishovite was similar to the one detected on unfractured as-grown quartz crystals, which were characterized only by H-bonded silanols on well-terminated surfaces (Pavan et al., 2020; Pavan et al., 2022). This silanol configuration is likely energetically unfavored in the interaction with membrane components. Consistently with this SAR hypothesis, both stishovite and as-grown quartz crystals were found non-membranolytic and non-cytotoxic to macrophages (Fenoglio et al., 2000; Pavan et al., 2020). As clarified in computational studies, the nature of the H-bonds and van der Waals forces formed at the silica surface outlines the main adsorption properties of silica. A strong intersilanol H-bond defines a silica surface that is less prone to interact with external molecules, because of a higher energy cost required to break pre-existing H-bonds (Musso et al., 2011; Musso et al., 2017). The peculiar silanol configuration of stishovite may also be the reason why this mineral exhibits the less negative surface charge with respect to the other polymorphs (−19 mV vs. −27 mV of quartz, Table 1). Strong H-bonded silanols might exhibit a lower Brønsted acidity than weakly interacting silanols, including NFS and isolated, in which the bonded hydrogen might show a more acidic character. This is in agreement with molecular dynamic simulations that identified out-of-plane silanols with a strong acidic character (pKa = 5.6) and in-plane silanols, e.g., silanols that belong to a network of intersilanol H-bonds, that do not yield high acidity (pKa = 8.5) (Leung et al., 2009; Sulpizi et al., 2012). We exclude that the mere particle morphology plays a role in membrane interaction. Indeed, coesite and stishovite showed similar roundish and ultrafine particles, but they demonstrated opposite membranolytic activity. Moreover, other silica materials characterized by curved and smooth particles, such as pyrogenic silica, have been proven to be highly membranolytic (Zhang et al., 2012).

Thus, overall, the differences in membranolytic activity observed among polymorphs may be ascribed to differences in the nature of the surface exposed and, specifically, to the occurrence of NFS. The crystal structure should partially determine the silanol distribution and intersilanol H-bonding properties of the various polymorphs. Polymorphs with an open crystal lattice, i.e., quartz, cristobalite, and tridymite, show patches of weakly interacting silanol groups in addition to networks of regular H-bond chains, as also reported in computational studies (Musso et al., 2009; Musso et al., 2017). However, in a real scenario, NFS might also be due to mechanical fragmentation and/or thermal treatments used to obtain the particles from raw materials. The structure of coesite is denser than quartz but NFS may still able to form on this polymorph (Wiessner et al., 1988). On the other hand, stishovite, that exhibits the highest crystal packing density, doesn’t show the presence of any family of free or nearly-free silanol.

### 3.3 Tailoring nearly free silanols and membranolytic activity of cristobalite and stishovite

**FIGURE 3:**
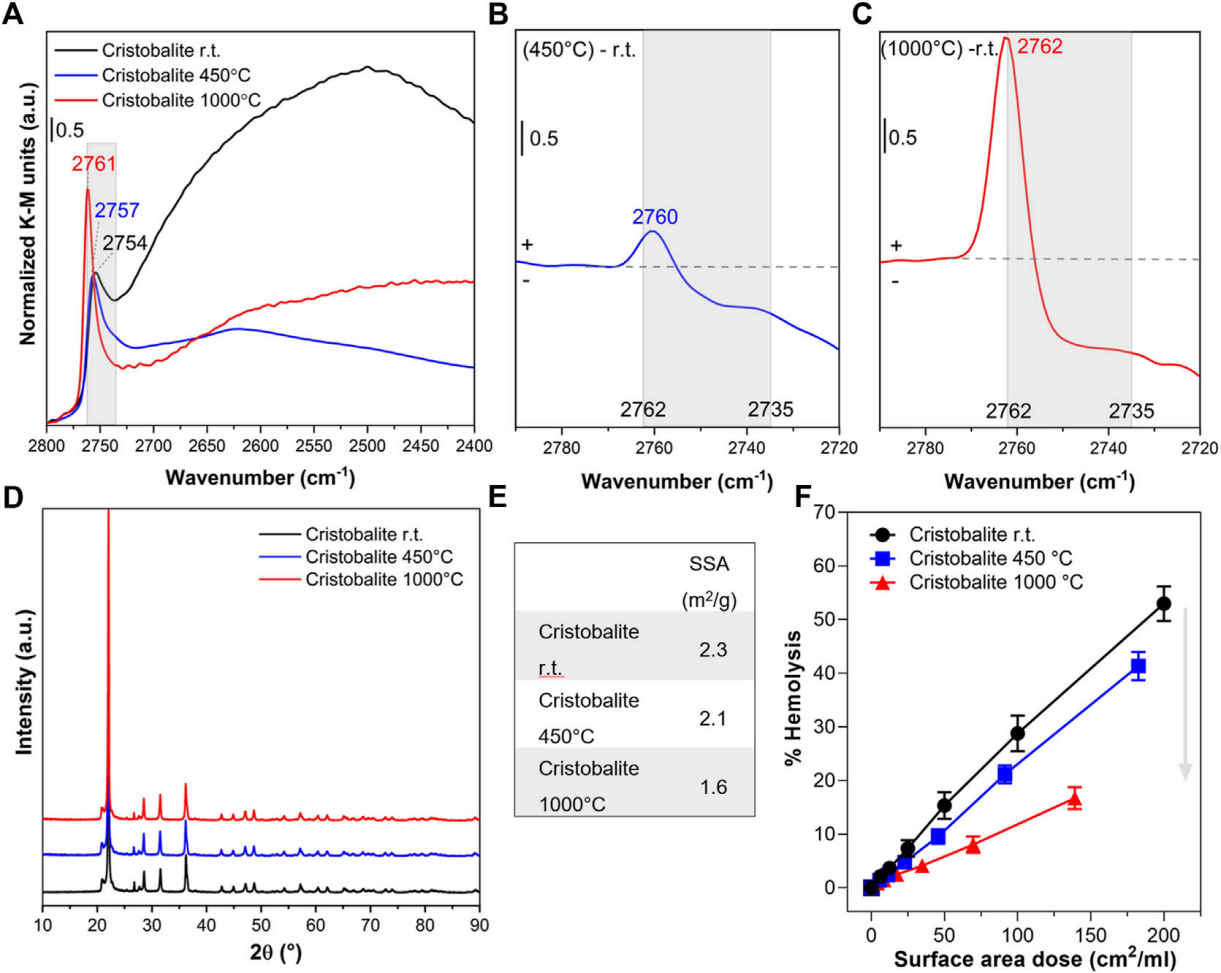
Nearly free silanols (NFS) determine the membranolytic activity of cristobalite. Pristine cristobalite particles (i.e., room temperature, r.t.) were calcined at 450° and 1,000°C. **(A)** Surface silanol distribution measured by DRIFT spectroscopy after H/D exchange in the νOD spectral region. **(B,C)** Differences in silanol population between cristobalite calcined at 450°C and pristine **(B)** or cristobalite calcined at 1,000°C and pristine **(C)**. Spectra are centered to the νOD spectral region assigned to NFS (2,760–2,735 cm^−1^) and isolated silanols. **(D)** XRPD patterns. **(E)** Specific surface area evaluated by BET method with Kr adsorption. **(F)** Membranolytic activity (percent hemolysis) of particles that were incubated for 30 min with purified sheep RBCs. Data are reported per surface area dose and are mean ± SEM of three independent experiments.

**FIGURE 4:**
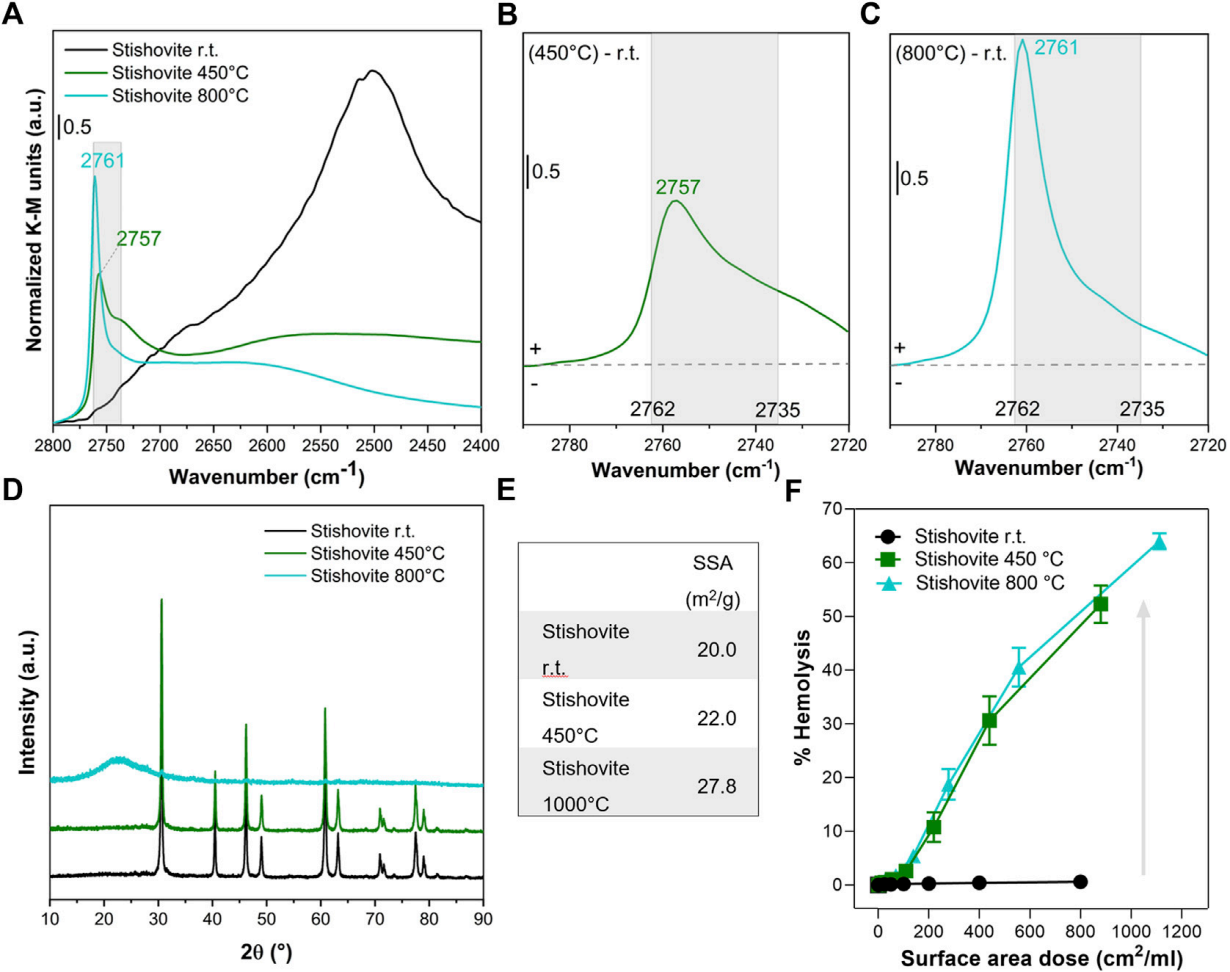
Nearly free silanols (NFS) determine the membranolytic activity of stishovite. Pristine stishovite particles (i.e., room temperature, r.t.) were calcined at 450° and 800°C. **(A)** Surface silanol distribution measured by DRIFT spectroscopy after H/D exchange in the νOD spectral region. **(B,C)** Differences in silanol population between stishovite calcined at 450°C and pristine **(B)** or stishovite calcined at 800°C and pristine **(C)**. Spectra are centered to the νOD spectral region assigned to NFS (2,762–2,735 cm^−1^) and isolated silanols. **(D)** XRPD patterns. **(E)** Specific surface area evaluated by BET method with N_2_ adsorption. **(F)** Membranolytic activity (percent hemolysis) of particles that were incubated for 30 min with purified sheep RBCs. Data are reported per surface area dose and are mean ± SEM of three independent experiments.

To confirm that NFS determine the membranolytic activity of the CS polymorphs, we performed thermal treatments to progressively tune the surface silanol patterns of cristobalite (Figure 3) and stishovite (Figure 4). These two polymorphs were selected because, among the other CS polymorphs: i. cristobalite deserves a relevant attention in the context of occupational human hazards, being classified as carcinogenic by the IARC; ii. stishovite shows peculiar structural (i.e., octahedral coordination of the Si atoms) and surface properties with respect to the other polymorphs (Cerrato et al., 1995), and resulted non-pathogenic in several *in vivo* and *in vitro* tests (Brieger and Gross, 1967; Driscoll, 1995).

Cristobalite particles were calcined at 450°C and 1,000°C. These temperatures were selected on the basis of previous investigations which demonstrated variation in the cytotoxic activity of this polymorph (Fubini et al., 1999). In particular, the cytotoxic effects towards macrophages and lung epithelial cells of cristobalite heated at temperatures above 800°C were reduced or completely suppressed. After calcination at 450°C, the major component assigned to silanols mutually engaged in strong H-bonds (from 2,720 to 2,400 cm^−1^) was irreversibly removed because of the condensation of these vicinal silanols into siloxane bridges (≡Si-OH + ≡Si-OH → ≡Si-O-Si≡ + H_2_O) (Figure 3A). As a consequence, a slight increase of the band related to the more distant/less interacting NFS was observed, as indicated by the upshift at 2,760 cm^−1^ of the νOD component at the highest frequency (Figure 3B). However, compared to what took place on quartz particles (Pavan et al., 2020), the increase of the peak at 2,760 cm^−1^ was very limited and compensated by a reduction in the NFS at lower frequency (2,745 cm^−1^). These data support the hypothesis that silanol condensation proceeds differently on cristobalite and quartz, as the different structure of the two polymorphs differently react to the same thermal treatment. Indeed, the more open structure of cristobalite with respect to quartz might generate a less favorable environment for silanol condensation. This is also consistent with the different condensation dynamics exhibited by quartz and amorphous silica (Pavan et al., 2020).

Calcination at 1,000°C resulted in the removal of a significant portion of the NFS detected between ca. 2,756 and 2,735 cm^−1^, with a further shift of the peak towards higher frequencies, where the vibrational mode of isolated silanols is detected (2,762 cm^−1^) (Figure 3C). Beside the changes in the mutual arrangement of surface silanols, the main structural features of cristobalite were not altered after the thermal treatments. Indeed, the XRPD pattern of cristobalite was not affected at the temperature of the treatments (Figure 3D). The positions, intensity ratios, and the line broadening of diffraction peaks were largely unaltered after both thermal treatments. The low SSA of cristobalite (Figure 3E) was slightly reduced only by heating at 1,000°C, possibly because of a limited sintering of the smaller particles into larger aggregates.

The possible relationship between surface silanol populations and the membranolytic activity of the pristine and calcined cristobalite samples was investigated by comparing dose-response curves of the three samples (Figure 3F). After calcining at 450°C, the dramatic reduction in the abundance of H-bonded silanols resulted in a negligible modification of the membranolytic activity of cristobalite, likely because the total amount of NFS exposed at mineral surface remained virtually the same. In contrast, after calcination at 1,000°C, the membranolytic activity was drastically reduced and that paralleled the strong reduction of NFS detected by FTIR. This finding supports the relevance of NFS as key mediator of the membranolytic activity not only of quartz but also of cristobalite.

We also calcined stishovite at a mild (450°C) or high (800°C) temperature to induce the selective modification of the silanol pattern (Figures 4A–C). After heating at 450°C, the most vicinal/interacting surface silanols promptly condensed (Cerrato et al., 1995) and the νOD vibrational features shifted from the region assigned to the strongly towards the weakly interacting silanols. In particular, the strong band at ca. 2,500 cm^−1^ drastically reduced towards a broadish adsorption in the 2,500–2,700 cm^−1^ range (Figure 4A), while the peak of NFS (2,757 cm^−1^) clearly emerged from the partially overlapping bands of the other silanol families (Figure 4B). By heating at 800°C, the NFS band further increased in intensity, albeit narrowed and shifted towards the frequency of the isolated silanols (2,761 cm^−1^) (Figure 4C). The different structure (i.e., octahedral coordination of the Si atoms) and the metastable nature of stishovite may possibly explain the different surface dehydration pathways observed for stishovite and cristobalite, especially in terms of modulation of the NFS population. The crystallinity of the metastable stishovite was not significantly affected by heating at 450°C, as shift and/or broadening of diffraction lines were negligible (Figure 4D). However, a slight decrease of the peak intensities was observed. Following the treatment at 800°C, the XRPD pattern was markedly modified. A broad halo in the low 2θ region was observed and likely assigned to the formation of amorphous silica (Gazzano et al., 2012). The main diffraction peak due to stishovite was still visible on the XRPD pattern, suggesting the presence of a residual crystalline phase. The observed loss of stishovite crystal structure and transformation into an amorphous phase at thermal treatment >800°C was previously reported (Cerrato et al., 1995). Before collapsing into amorphous silica, stishovite structure should evolve from the octahedral to the typical tetrahedral Si coordination and a less rigid organization of the Si-O-Si network may occur. Moreover, the SSA progressively increased by increasing the temperature of the treatment, ranging from 20 to ca. 27 m^2^/g at 800°C.

The effect of the thermal treatments on the membranolytic activity of stishovite (Figure 4F) resulted in an opposite effect to that observed for quartz and cristobalite, albeit the increase in the membranolytic activity still reflected the formation of NFS on the heated stishovite. After treating stishovite at 450°C, the decrease of H-bonded silanols and the formation of NFS caused a strong increase of the membranolytic activity of the polymorph, which exhibited a 50% hemolysis at the highest dose tested. By further heating at 800°C, the membranolytic activity of stishovite wasn’t reduced, and the dose-response curve virtually overlapped the one obtained with the sample heated at 450°C. This paralleled the overall amount of NFS that are available in the two heated samples. Indeed, the high-frequency peak of 800°C-stishovite is narrowed and shifted towards the higher frequency values of isolated silanols, but the intensity of the IR signal in the NFS region is largely preserved. The low amount of alumina and titania that contaminate stishovite and the negligible membranolytic activity of these two oxides (Lu et al., 2009; Cho et al., 2013) clearly indicates that the membranolytic activity observed for calcined stishovite is due to the increase of NFS induced on stishovite surfaces.

Notably, even if the membranolytic activity didn’t vary and the surface silanol profile changed slightly from 450°C to 800°C, stishovite transformed from crystalline to amorphous structure. We might speculate that the amorphization process first (at 450°C) started from the surface, induced a disorganization of the surface silanol network not evidenced by XRPD analysis, and then continued up to the bulk at high temperatures (800°C). This would be in line with the notion that surface amorphization determines an irregular network of H-bonded silanols forming patches of NFS that are responsible with the silica-membrane interaction. This finding thus supports the notion that disorganization of the surface silanol network induced by fracturing, thermal treatments, or other processes that alter the crystal surface, creates reactive surface silanol patches that may have an impact on the stability of cell membranes.

## 4 Conclusion

The SAR between the surface properties of CS polymorphs and their capacity to induce cell membrane damage, which represents the molecular initiating event of the toxicity mechanism of silica particles (Pavan and Fubini, 2017), was here investigated. We demonstrated that the diverse crystal packing of the CS polymorphs creates different silanol networks on the surfaces, characterized by different amount of NFS. These differences in surface silanol topochemistry promotes variable recognition efficiency when silica is contacted with cell membranes. Overall, we showed that the specific family of NFS is responsible for the membranolytic activity of all CS polymorph. All CS polymorphs here investigated but stishovite, i.e., quartz, cristobalite, tridymite, and coesite, showed surface NFS and caused RBC membrane damage. Stishovite, due to its atypical octahedral coordination of silicon and higher surface silanol density (Cerrato et al., 1995), showed only strongly H-bonded silanols on its surface. By modulating the amount of NFS with thermal treatments, we demonstrated that the silica membranolytic activity positively correlates with the amount of NFS for all CS polymorph, including stishovite and amorphous silica resulting from lattice collapsing. We suggest here that, for CS polymorphs whose toxic activity is well established (Brieger and Gross, 1967; Driscoll, 1995; IARC, 2012), the membranolytic activity is NFS-mediated. Similarly, the negligible toxicity and membranolytic activity of pristine stishovite is related to the negligible amount of NFS that are exposed at the surface of this polymorph. This work on the membranolytic activity of CS polymorphs was limited to RBC membrane as a proxy for cell membrane, including phagolysosome membrane. Further studies with other simplified model membranes (e.g., liposomes and phospholipids) and surrogate lung cell models should aim to demonstrate the relevance of NFS for phagolysosome membrane damage and activation of inflammatory and fibrotic pathways of CS polymorphs, as it has been done for quartz (Pavan et al., 2020). Molecular modelling could also be used to increase the comprehension of the SAR between surface topochemistry of CS polymorphs and the membrane epitopes that are molecularly involved in the bio-inorganic interaction.

Overall, these findings contribute to the molecular understanding of the toxicity mechanism of silica-based minerals, and might be helpful for predicting and controlling the hazard associated to quartz and cristobalite, which are included in the IARC classification of human carcinogens (IARC, 2012; Cullinan et al., 2017). The other polymorphs, including tridymite, coesite, and stishovite, are less frequently found in nature, thus the relevance of their impact on human health is minor. Nonetheless, deposits of terrestrial and celestial rare CS polymorphs were recently reported (Roskosz and Leroux, 2015; Morris et al., 2016; Kayama et al., 2018), hence our study might help to evaluate the hazard of also these rare CS polymorphs in future exploration and possible exploitations. Finally, the comprehension of the surface characteristics and reactivity of CS polymorphs here described could provide further hints for explaining complex bio-mineral interfacial phenomena, including prebiotic chemistry reactions (Rimola et al., 2018; Rimola et al., 2019) and a variety of heterogeneous atmospheric processes (Sihvonen et al., 2018).

## Data Availability

The raw data supporting the conclusion of this article will be made available by the authors, without undue reservation.
