# Supplementary material for: Nearly free silanols drive the interaction of crystalline silica polymorphs with membranes: Implications for mineral toxicity
Source: Front Chem. 2023 Jan 16;10:1092221. doi: 10.3389/fchem.2022.1092221 (PMC9884702; doi:10.3389/fchem.2022.1092221)
Supplement: Supplementary file 1 [file DataSheet1.pdf]

Supplementary Material

Nearly free silanols drive the interaction of crystalline silica polymorphs with membranes: Implications for mineral toxicity

Cristina Pavan[1,2,3], Guilllermo Escolano-Casado[1,4], Chiara Bellomo[1,2], Stefania Cananà[1,2], Maura Tomatis[1,2], Riccardo Leinardi[3], Lorenzo Mino[1,4], and Francesco Turci[1,2*]

[1]Department of Chemistry, University of Turin, Turin, Italy

[2]"G. Scansetti" Interdepartmental Centre for Studies on Asbestos and Other Toxic Particulates, University of Turin, Turin, Italy

[3]Louvain Centre for Toxicology and Applied Pharmacology, Institute of Experimental and Clinical Research, Université catholique de Louvain, Brussels, Belgium

[4]Nanostructured Interfaces and Surfaces Interdepartmental Centre, University of Turin, Turin, Italy

* Correspondence:

Prof. Francesco Turci

francesco.turci@unito.it

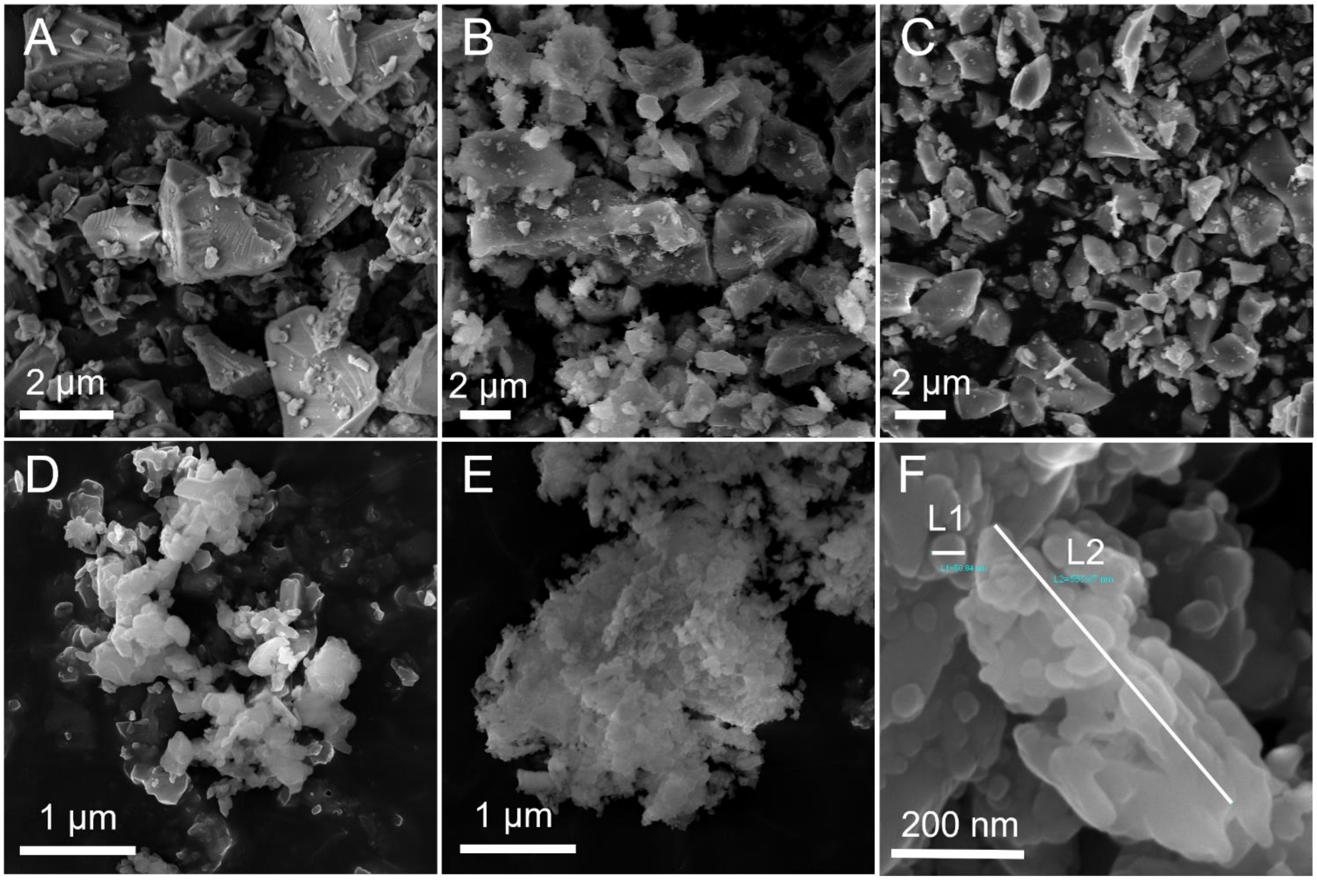

Supplementary Figure S1. Morphology of CS polymorphs obtained by FESEM. Low magnification micrographs: (A) quartz, (B) cristobalite, (C) tridymite, (D) coesite, and (E) stishovite particles. (F) High magnification of stishovite showing primary particles in nanometric size (L1: 50 nm) and larger aggregates/agglomerates (L2: 555 nm).

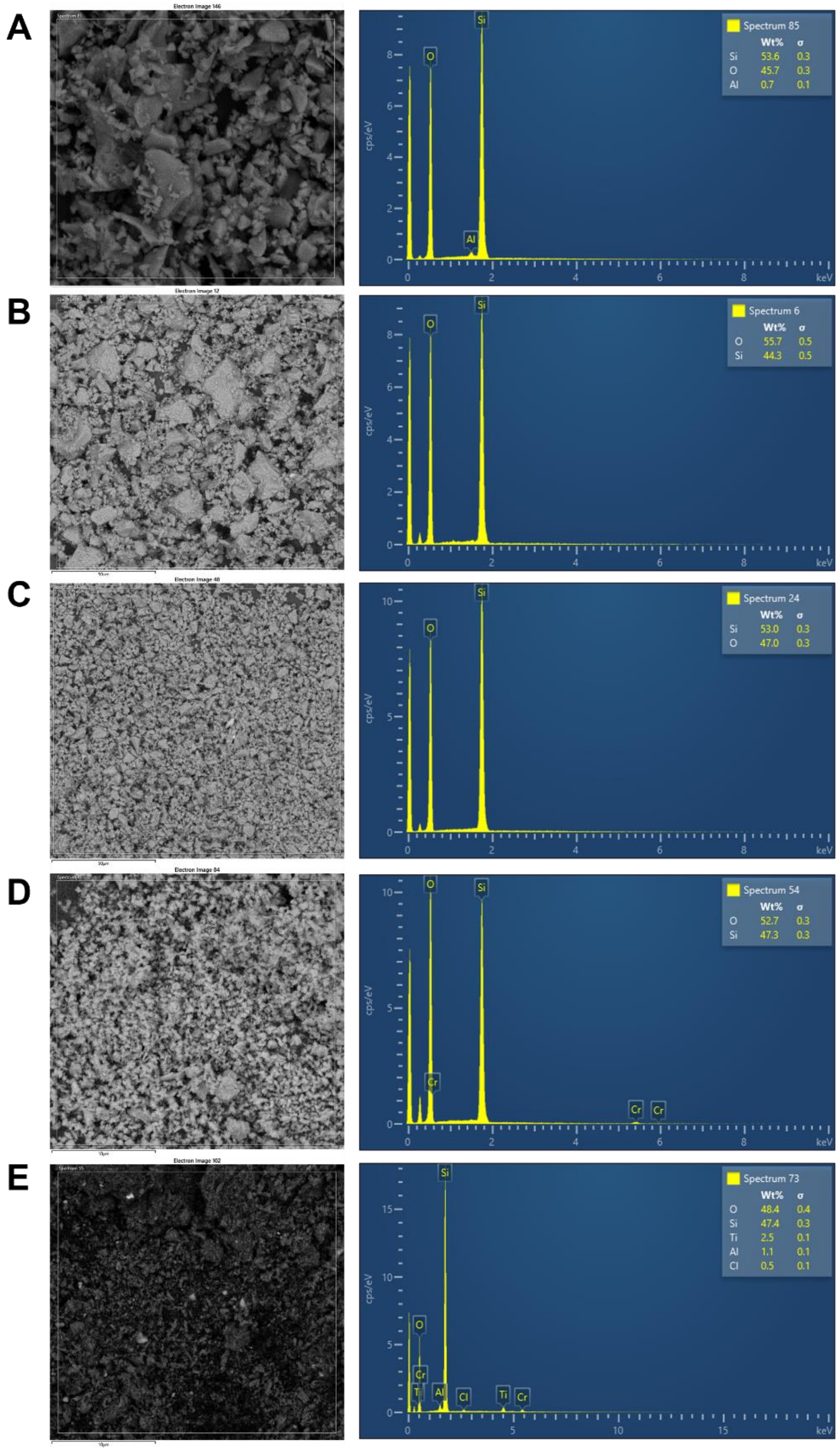

Supplementary Figure S2. Spectra obtained by EDX analysis of one sample region (ca. 650 μm^2) of (A) quartz, (B) cristobalite, (C) tridymite, (D) coesite, and (E) stishovite particles. At least twelve regions were collected per sample, at 2-10k magnification, 10 or 20 KV accelerating voltage, and processed using Aztec suite (v. 4.2, Oxford Instruments, UK) to obtain the average values reported in Table 1. Occurrence of chrome is related to particle coating to prevent the electron beam from charging the sample during the analysis.

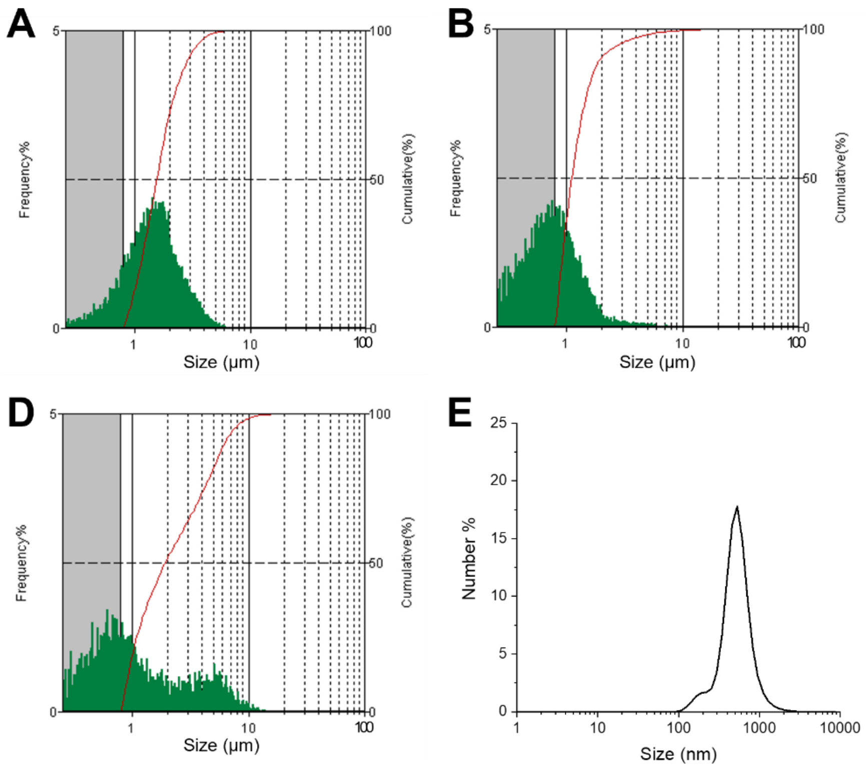

Supplementary Figure S3. Particle size distribution curves of CS polymorphs dispersed in medium used for hemolysis test. Frequency and cumulative percentage of all measured particles dispersed in 0.01M PBS (1 mg/ml) obtained by FPIA analysis: (A) quartz, (B) cristobalite, (C) tridymite, and (D) stishovite particles. Below 0.8µm (the lowest detection limit) the measure is not statistically relevant even if particles are detected. (E) Size distribution (number %) of coesite particles dispersed in 0.01M PBS (0.1 mg/ml) and measured by DLS analysis. Only for coesite dispersion the quality of the DLS measure was good, Z-average of 0.70 µm ± 0.12 (PdI: 0.23).

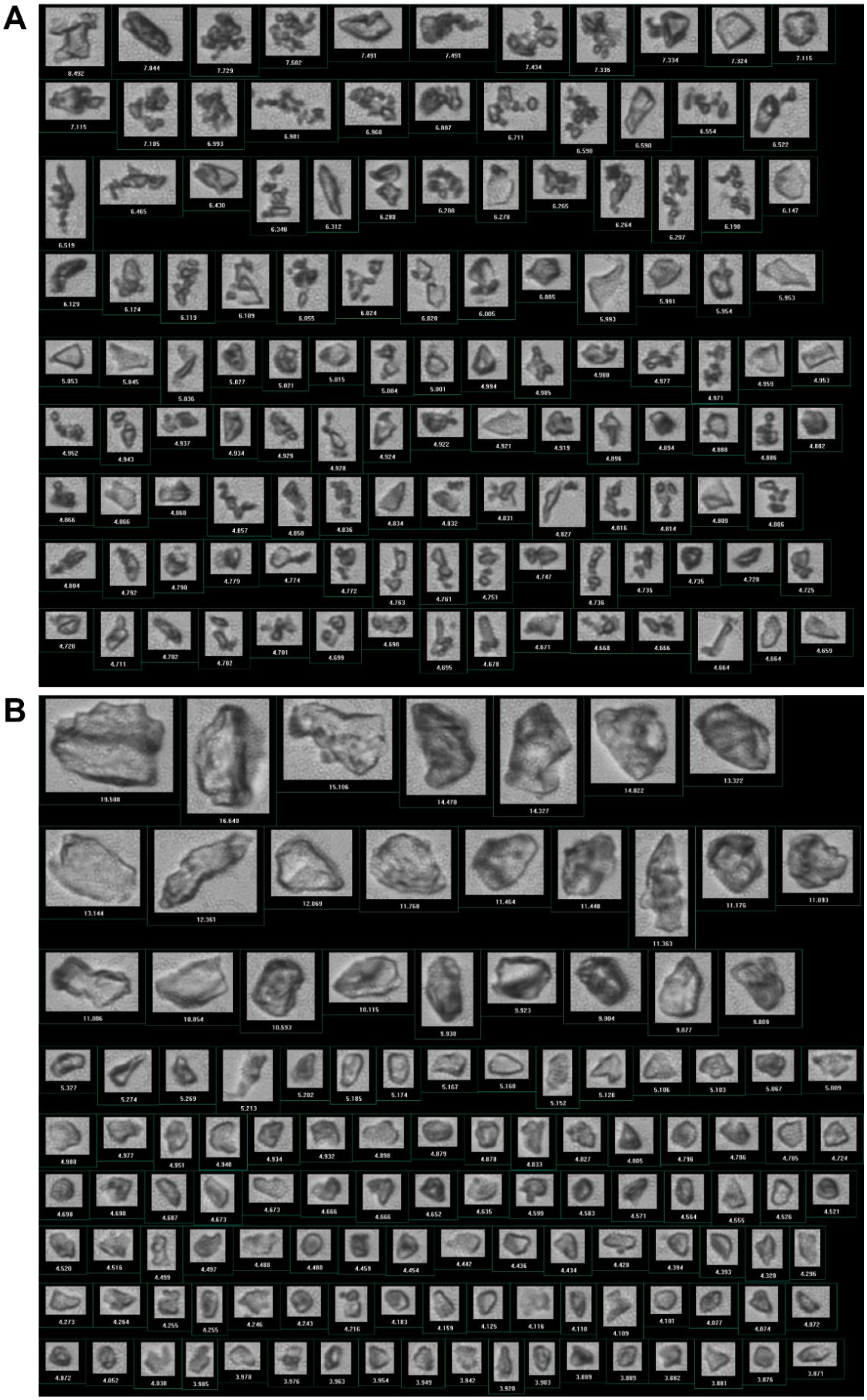
A
B

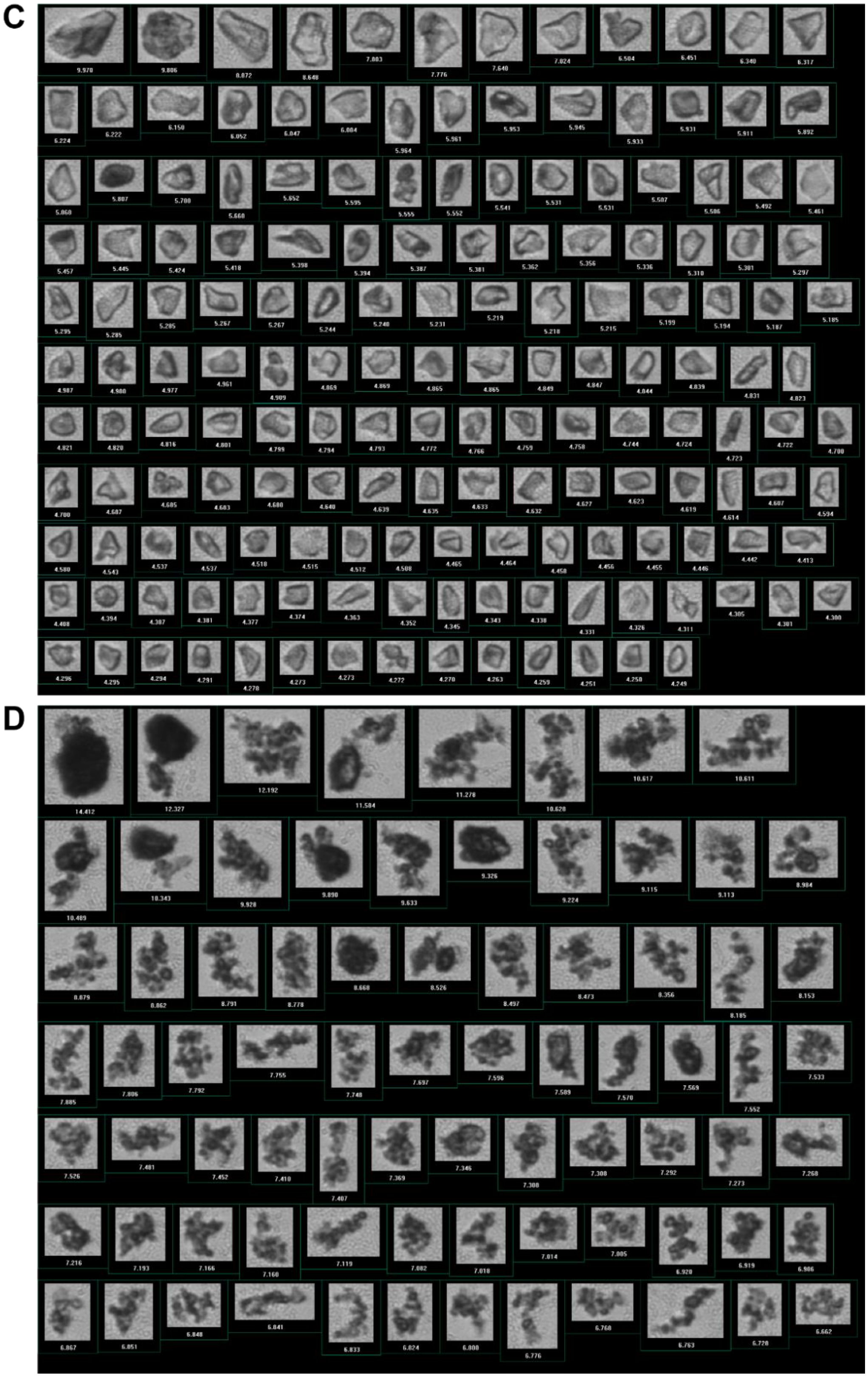

Supplementary Figure S4. Images of the particles captured by FPIA in 0.01M PBS: (A) quartz, (B) cristobalite, (C) tridymite, and (D) stishovite particles. Particles are listed in descending order CE diameter

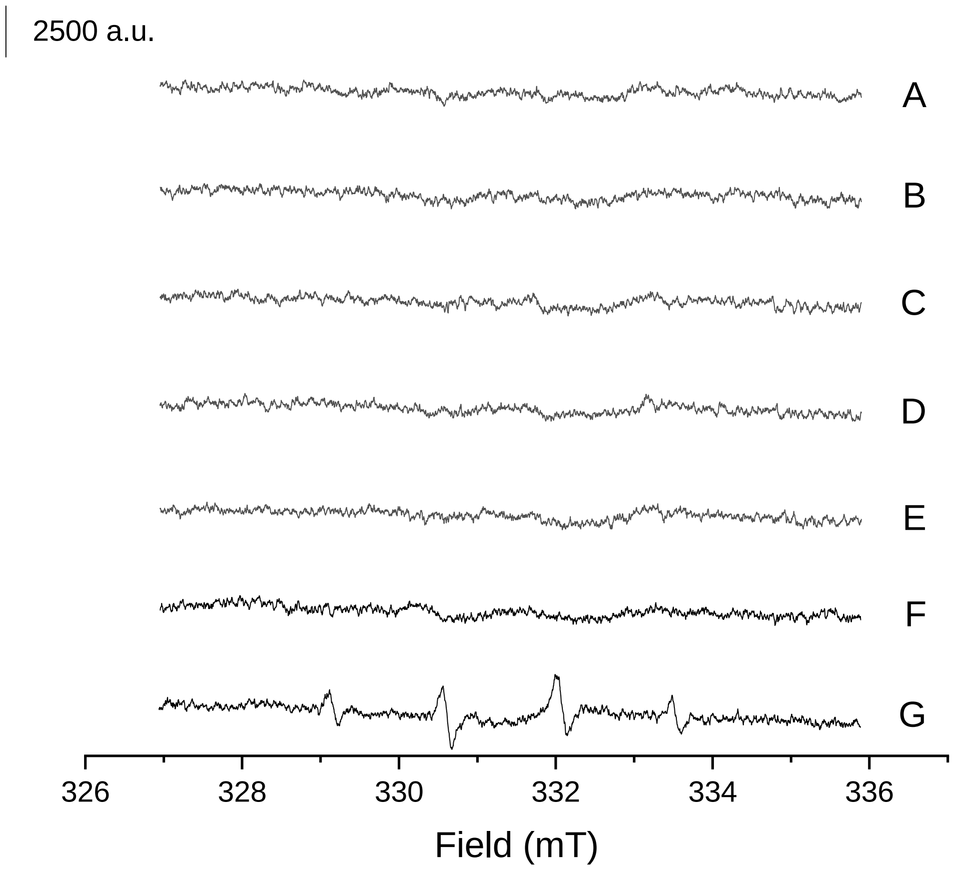

Supplementary Figure S5. Hydroxyl radical (•OH) generation measured by EPR spectroscopy coupled with the spin trapping technique after incubation of the CS polymorph samples for 15 min in DMPO: (A) quartz, (B) cristobalite, (C) tridymite, (D) coesite, (E) stishovite, (F) blank (same experimental conditions without the samples), and (G) TiO_2 nanopowder (P25) used as positive reference particle.

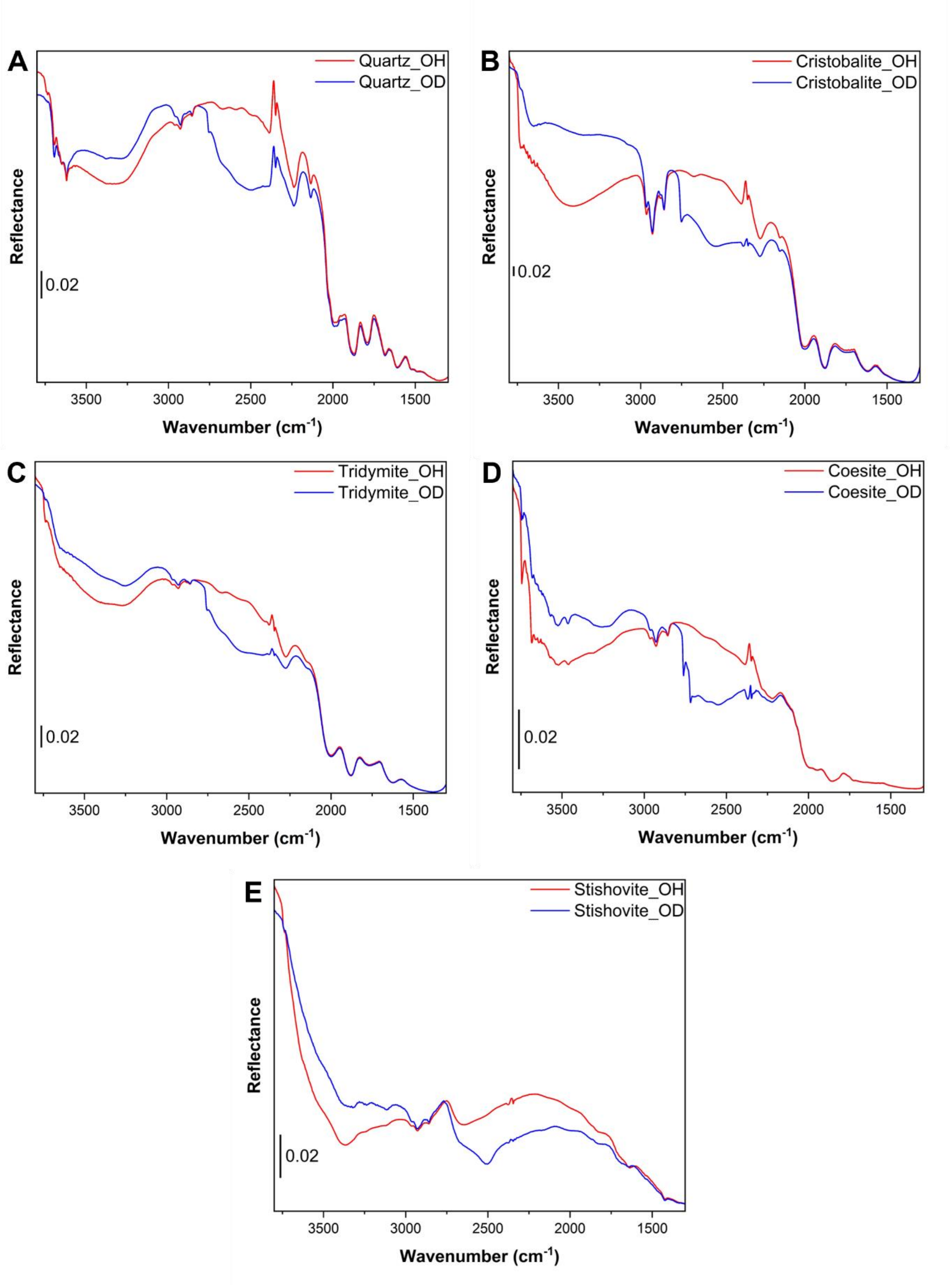

Supplementary Figure S6. Reflectance spectra before and after isotopic exchange with D_2O for all the CS polymorphs: (A) quartz, (B) cristobalite, (C) tridymite, (D) coesite, and (E) stishovite particles.
